# Mitotype Interacts With Diet to Influence Longevity, Fitness, and Mitochondrial Functions in Adult Female *Drosophila*

**DOI:** 10.3389/fgene.2018.00593

**Published:** 2018-11-30

**Authors:** Samuel G. Towarnicki, J. William O. Ballard

**Affiliations:** School of Biotechnology and Biomolecular Sciences, University of New South Wales, Sydney, NSW, Australia

**Keywords:** *Drosophila*, mitotype, diet, mitochondria – DNA, OXPHOS = oxidative phosphorylation

## Abstract

Mitochondrial DNA (mtDNA) and the dietary macronutrient ratio are known to influence a wide range of phenotypic traits including longevity, fitness and energy production. Commonly mtDNA mutations are posited to be selectively neutral or reduce fitness and, to date, no selectively advantageous mtDNA mutations have been experimentally demonstrated in adult female *Drosophila*. Here we propose that a ND V161L mutation interacted with diets differing in their macronutrient ratios to influence organismal physiology and mitochondrial traits, but further studies are required to definitively show no linked mtDNA mutations are functionally significant. We utilized two mtDNA types (mitotypes) fed either a 1:2 Protein: Carbohydrate (P:C) or 1:16 P:C diet. When fed the former diet, Dahomey females harboring the V161L mitotype lived longer than those with the Alstonville mitotype and had higher climbing, basal reactive oxygen species (ROS) and elevated *glutathione S-transferase E1* expression. The short lived Alstonville females ate more, had higher walking speed and elevated mitochondrial functions as suggested by respiratory control ratio (RCR), mtDNA copy number and expression of *mitochondrial transcription termination factor 3*. In contrast, Dahomey females fed 1:16 P:C were shorter lived, had higher fecundity, walking speed and mitochondrial functions. They had reduced climbing. This result suggests that mtDNA cannot be assumed to be a strictly neutral evolutionary marker when the dietary macronutrient ratio of a species varies over time and space and supports the hypothesis that mtDNA diversity may reflect the amount of time since the last selective sweep rather than strictly demographic processes.

## Introduction

“It’s better to burn out than fade away”

– Neil Young

Evolutionary biologists have long sought to understand the evolutionary forces that influence genetic variation within and among populations. At the molecular level polymorphisms might be evolving neutrally, could be transient variants on their way to elimination because they are deleterious, on their way to fixation because they are beneficial, or they are actively maintained by balancing selection. It is now well documented that strong purifying selection affects variability of mitochondrial DNA (mtDNA) encoded genes and the purging of deleterious variants will result in the removal of linked variants through background selection. Evidence of positive selection on mitogenomes has been reported (James et al., 2016; Rollins et al., 2016), but no specific mutation has been experimentally shown to have an evolutionary advantage in nature. In Pacific salmon, it has been proposed that changes in the mtDNA-encoded proton-pumping piston arm of Complex I influences organismal fitness (Garvin et al., 2015). In humans, the frequencies of mtDNA-encoded T3394C and G7697A are higher in a Tibetan high-altitude group compared with a low-altitude group and it is suggested that the mitochondrial genome might be under selection from the high-altitude hypoxic environment (Li et al., 2016).

In this study, we explore how diet affects the physiology and the mitochondrial functions of adult female *Drosophila melanogaster* harboring distinct mtDNA haplotypes (mitotypes). Historically, caloric restriction was thought to reduce mitochondrial energy output and cellular damage which slowed the aging process (Partridge et al., 2005). More recent studies have shown that it is the macronutrient source of the calories rather than the total amount that leads to differential longevity (Lee et al., 2008; Solon-Biet et al., 2014; Zhu et al., 2014; Solon-Biet et al., 2015). Diets high in protein are correlated with reduced longevity compared to diets high in carbohydrates (Lee et al., 2008). Specifically, the ratio of macronutrients in diet was found to be the causative factor that increased risk of death. Protein: carbohydrate ratios (P:C) higher than 1:2 P:C (such as 1:1 or 2:1) had the greatest risk of death, while diets with a low P:C ratio (such as 1:16 P:C) had the lowest risk of death. Here, we investigate physiological traits and mitochondrial functions of flies fed macronutrient ratio’s of 1:2 P:C and 1:16 P:C. These ratio’s span the macronutrient range fed upon by *Drosophila* in nature.

We measured fecundity and feeding using the CAFÉ assay (Lee et al., 2008). Fecundity is energetically expensive (Melvin and Ballard, 2011) and there is a trade-off with longevity (Westendorp and Kirkwood, 1998; Lee et al., 2008; Correa et al., 2012; Solon-Biet et al., 2014) that is modified by an organism’s genes and the environment (Beaulieu et al., 2015). While this trade-off is seen to be important in longer lived organisms such as humans (Kaptijn et al., 2015) it has also been shown to occur in comparatively shorter lived organisms including insects (Haeler et al., 2014; Zhu et al., 2014). CAFÉ feeding rate was measured as it provides a robust measure of food consumption. Feeding rate has been shown to differ between mitotypes in adult males and in female larvae (Pichaud et al., 2013; Towarnicki and Ballard, 2017). We measure the expression of *Notch* (*N*) to gain a cellular link with feeding rate. Upregulation of *N* has been shown to block CREB (Zhang et al., 2013), and lead to an increased feeding response in *Drosophila* (Iijima et al., 2009).

We hypothesized that there may be physiological trade-offs between lifespan and other energetically expensive process such as physical activity. Walking speed and climbing ability were assayed as measures of physical activity as these provide different measures of energy expenditure. We employ average walking speed as a measure of basal physical activity. In contrast, we include the climbing assay as a measure of short-term explosive energy that follows physical disturbance. Walking speed has been shown to correlate with lower survival in insects (Ragland and Sohal, 1975; Melvin and Ballard, 2011). Climbing tends to decreases with age in *Drosophila* (Goddeeris et al., 2003; Gargano et al., 2005).

As measures of mitochondrial function, we assayed the respiratory control ratio (RCR), mtDNA copy number and *mitochondrial transcription termination factor 3* (*mTerf3*) expression. RCR indicates the ability to make ATP. A high RCR occurs when mitochondria respond to the addition of ADP, followed by a fast return to basal levels when coupled (Brand and Nicholls, 2011). In healthy individuals, copy number is an indirect measurement of the OXPHOS capability of an organism, and may indicate maintenance of mitochondrial health (Dickinson et al., 2013). *mTerf3* is the primary regulator of mitochondrial biogenesis and we hypothesized its expression may be correlated with copy number (Nam and Kang, 2005; Roberti et al., 2006, 2009).

We assayed reactive oxygen species (ROS) production and an aspect of the antioxidant response as potential indicators of mitohormesis. Mitohormesis is defined as a non-linear response to ROS, where low levels of ROS benefit lifespan, but high levels are detrimental (Ristow, 2014). Here we assayed basal ROS levels from extracted mitochondria. Mitochondria are the main source of ROS production and their function is strongly influenced by mitotype and diet (Ballard, 2005; Bellizzi et al., 2012; Sun et al., 2012; Yu et al., 2015; Aw et al., 2017). Under mitohormesis low levels of ROS act as signaling molecules that provides a fitness advantage through higher antioxidant capacity (Tapia, 2006; Ristow and Schmeisser, 2014). However, high ROS levels are predicted to reduce longevity primarily through DNA damage, but also through damage of proteins and fats. We assay expression of *Glutathione S-transferases E1* (*GstE1*) as a measure of the antioxidant response (Sharma et al., 2004). GSTE’s are a family of enzymes that detoxify lipids hyperoxides that result from ROS damage (Sheehan et al., 2001).

We included two *Drosophila* mitotypes throughout this study, Alstonville and Dahomey. Each of these mitotypes was genetically placed into two standard nuclear genetic backgrounds to test the generality of the mtDNA effects. Mitonuclear interactions have previously been shown to influence longevity and fitness (Clancy, 2008; Parmakelis et al., 2013; Wolff et al., 2014; Zhu et al., 2014). The Alstonville and Dahomey mitotypes differ by non-synonymous mutations in ND4 (V161L), ATP6 (M554I), and COXIII (D117N). They also differed by silent mutations in the lrRNA (G497A), the srRNA (A78U and A240G) and have 52 differences in the A+T rich region. Aw et al. (2018) studied these same mitotypes and observed a diet specific flip in larval development time that they argued was caused by the ND4 (V161L) mutation in Dahomey. When fed the 1:2 P:C food Dahomey larvae developed slower. However, when larvae were fed 1:16 P:C food metabolism was extensively remodeled and larval development time was shorter in Dahomey than Alstonville. Quaternary structure modeling posited that the ND4 (V161L) mutation reduced proton pumping and did not interact with any nuclear encoded subunits. Given this same suite of mutations occurs in all life history stages our prediction was that ND4 (V161L) mutation is also functional in adults. We hypothesize that reduced proton pumping in Dahomey females will decrease OXPHOS and increase longevity at the expense of reduced fecundity and basal physical activity (Copeland et al., 2009).

In this study we show that diet interacts with the host mitotype to influence longevity. We provide experimental evidence showing that adult females harboring the Dahomey mitotype are longer lived than Alstonville flies on a high protein 1:2 P:C diet but shorter lived on a high carbohydrate 1:16 P:C diet. This flip in longevity is inversely correlated with mitochondrial functions and is independent of the nuclear genetic background. This result has important implications in the field of molecular ecology as it supports the hypothesis that mtDNA diversity may reflect the amount of time since the last selective sweep (fixation of one haplotype as a result of the fitness advantage of one or more of its component nucleotides) rather than strictly demographic processes affecting the population (Ballard and Whitlock, 2004; James et al., 2016).

## Materials and Methods

### Fly Strains and Maintenance

The five fly strains were used in this study were constructed from three mitotypes and two nuclear DNA backgrounds. We refer to the three mitotypes as Alstonville, Dahomey and *w*^1118^. The former two were introgressed into the *w*^1118^ and Oregon R nuclear genetic backgrounds using balancer chromosomes followed by at least five generations of backcrossing (Clancy, 2008; Towarnicki and Ballard, 2017). To reduce the incidence of accumulated nuclear mutations, females from all fly strains were backcrossed to males of their corresponding nuclear genome for a minimum of five generations before all assays. In the coding region, the *w*^1118^ mitotype differs from Alstonville and Dahomey mtDNA by six and nine non-synonymous mutations, respectively (Clancy, 2008). To verify the correct strains were used, flies were genotyped at the beginning and end of each assay using allele specific PCR (Aw et al., 2018). Amplicons were run on a 1% agarose gel, with a band indicating Alstonville mtDNA, and no band Dahomey mtDNA.

Stock flies were maintained at constant density of 200 ± 25 adults in 250 ml glass bottles on instant *Drosophila* media (Formula 4-24^®^ Instant *Drosophila* Medium, Plain, Carolina Biological Supply Company) at 23°C, 50% relative humidity with 12:12 light: dark cycles. To produce experimental flies, eggs were collected from stock flies using 5% agar, 10% treacle plates with 5 mm thick yeast paste. Eggs were collected, cleaned and placed on instant *Drosophila* medium following Clancy and Kennington (2001). After 2 days, four adult males of each mitotype were homogenized in 1.4 ml of ddH_2_O and 130 μl of the resulting solution was added to each bottle to standardize the microbiome of the larvae.

Mated adult female flies were included throughout the study. Females were sorted on ice 48 h after eclosion and then either placed into 500 ml demography cages, 5 ml CAFÉ assay vials or 25 ml vials, such that the density of flies in each container were similar. Male flies were not used in this study because we were interested in the trade-offs with egg production. Food vials were replaced every 2 days for each assay. Assays were carried out on flies that spent 12 days on either the 1:2 or 1:16 P:C diets. Previous studies have shown that bioenergetic and physiological assays of young flies were a good predictor of longevity (Melvin and Ballard, 2011). Assaying flies at 14 days of age ensured that no larval fat reserves remained (Aguila et al., 2013) and was before flies started dying in the longevity studies conducted here.

### Experimental Diets

Yeast-sugar diets were used in this study and were manipulated to have P:C ratios of 1:2 or 1:16, whilst remaining isocaloric. The 1:2 P:C diet comprised of 71.55 g of sucrose and 108.45 g of yeast per L at a final concentration of 180 g/L. The 1:16 P:C diet comprised of 157.4 g of sucrose and 22.6 g of yeast per L at a final concentration of 180 g/L.

### Longevity

In this study, 2 days old female *Drosophila* were added to 500 ml demography cages. Each cage contained 40 flies. Cages were randomly assigned to positions within the incubator and were moved every 2–3 days. Dead flies were counted and removed every 2–3 days when food was replaced. Flies that died in the first 6 days were not counted as these were considered to be related to handling. Two independent longevity assays were conducted with each mitotype in the *w*^1118^ and the Oregon R genetic backgrounds fed each macronutrient ratio. Each study had 120 flies/mitotype/genetic background/diet. No significant block effects were detected and the studies were pooled such that 240 flies/mitotype/genetic background/diet were included. We did not determine longevity using the CAFÉ assay because of the shorter survival period when flies are fed capillaries (Bajracharya and Ballard, 2016).

### CAFÉ Assay

The CAFÉ assay was used to measure fecundity and feeding simultaneously. In this study, females mated to males with same mitotype were individually transferred to 5 ml vials containing 500 μl of 1% agar. Capillary feeding tubes containing 5 μl of food were added to each vial and a no-fly control. Flies were transferred to new vials with new capillary feeding tubes daily. No flies died during the 14 days assay period.

#### Early Fecundity

Egg production is energetically expensive in *Drosophila* and early fecundity has been shown to reduce lifespan (Khazaeli and Curtsinger, 2013). Here, number of eggs laid by each female mated to a male of the same mitotype was counted daily until 14 days of age when the physiological and biochemical assays were conducted. To test the influence of male mitotype on female fecundity we also mated virgin females of each mitotype to *w*^1118^ males and calculated egg production. Egg production for each female was averaged because we were interested in the overall production/female. A total of 10 flies/mitotype/diet were assayed.

#### Feeding

Capillary feeding was assayed as a measure of food consumption. The volume of food eaten by each fly was recorded daily until 14 d of age and consumption averaged for each individual. Over this period there was a slight decline in daily food consumption that was not mitotype or diet specific. A total of 10 flies/mitotype/diet were assayed.

*N* expression was assayed as it is linked with a feeding response (Iijima et al., 2009) using *N* forward 5′-CGCTTCCTGCACAAGTGTC-3′, *N* reverse 3′-GCGCAGTAGGTTTTGCCATT-5′ (Hu et al., 2013). Qualitative real-time PCR was conducted using Sybr-Green chemistry following (Correa et al., 2012). RNA was extracted from 5 flies per sample. Following Aw et al. (2018) data for all gene expression assays was normalized to nuclear housekeeping genes *Actin* and *RP49* and expressed as relative expression to Alstonville within each diet. A total of 6 replicates/mitotype/diet were assayed for each treatment.

### Physical Activity

Two assays of physical activity were conducted. Walking speed was measured as an indicator of basal energy expenditure (Melvin and Ballard, 2011). Climbing ability is a rapid movement response and is a known indicator of senescence (Gargano et al., 2005).

#### Walking Speed

Activity was measured with the Trikinetics Activity monitor following Melvin and Ballard (2011). Briefly, experimental diet was poured in a beaker to a depth of 2–2.5 cm. Glass tubes were positioned vertically in the beaker and the end of the vial containing food was covered with a cap. One female was added to each activity tube and the vials were plugged with cotton wool to confine the fly to a distance of 4.5 cm in the tube and added to the holders of the activity monitor. Flies were allowed 12 h to acclimate before recording began at the flies “dawn.” The number of times a fly crossed the infrared beam at the midpoint of the tube was recorded by the DAM software (Trikinetics, Waltham, MA). Walking speed was calculated as: (number of light beam crossings × 4.5 cm)/12 h. When a fly was reported to cross the light beam more than 30 times in 5 min, the data point was excluded. A total of 16 flies/mitotype/diet were assayed.

#### Climbing Assay

We assayed climbing ability following Bajracharya and Ballard (2016). Flies were transferred to vials without food and allowed to recover for 1 h. Vials were randomly grouped in lots of six, tapped three times to knock flies to the base of the vial and then photographed after 4 s by a camera 50 cm away. The number of flies that climbed above the 80 mm mark of the vial was recorded. A total of six vials of 10 adult flies/treatment were assayed.

### Mitochondrial Functions

To test whether changes in longevity involved differences in mitochondrial energy metabolism we assayed RCR, mtDNA copy number, *mTerf3* expression, basal ROS and one aspect of the antioxidant response.

#### RCR, Copy Number, and *mTerf3* Expression

We quantified RCR as an estimate of mitochondrial health. Mitochondria were isolated from 10 flies per mitotype. Following Aw et al. (2016), RCR was measured by Seahorse XF 24 as state III respiration in the presence of ADP and substrate, over basal state IVo respiration. The XF sensor cartridge was loaded with 4 injection compounds (ADP/oligomycin/BAM15/rotenone and antimycin A). Each plate was visualized under the microscope to ensure a monolayer of adhered mitochondria to the well bottom. A total of 6 replicates/mitotype/diet were assayed.

MtDNA copy number was measured as an indicator of OXPHOS capacity. Copy number was determined following Aw et al. (2018). A total of 6 replicates/mitotype/diet were assayed for each treatment.

*mTerf3* was assayed as a measure of mitochondrial transcription that we hypothesized may be linked with copy number. Primer pair: *mTerf3* forward 5′-TAACATCACCGGGTATAACCACC-3′, reverse 3′-CACTTCTTTGGAGCCTTCACAT-5′ (Hu et al., 2013). Data from all gene expression assays were normalized to the nuclear housekeeping genes *Actin* and *RP49* and expressed as relative expression to Alstonville within each diet (Aw et al., 2018). A total of 6 replicates/mitotype/diet were assayed.

#### Basal ROS and Antioxidant Response

Low levels of basal ROS can increase longevity but above a threshold the benefit is lost and longevity declines (Ristow and Schmeisser, 2014). Mitochondria were isolated from 10 flies per mitotype and Amplex Red assay was undertaken following Melvin and Ballard (2006). A total of 6 replicate s/mitotype/diet were assayed.

We measured one aspect of the antioxidant response by quantifying the expression of *GStE1* (Vontas et al., 2001) following Aw et al. (2018). Again, *Actin* and *RP49* were the house keeping controls. A total of 6 replicates/mitotype/diet were assayed for each treatment.

### Statistics

We describe differences between the mitotypes relative to Dahomey because the ND4 mutation, which is predicted to be functionally important, occurs in this mtDNA (Aw et al., 2018). Longevity was calculated by the Log-Rank test using PRISM 7 (GraphPad Software). All other data were analyzed for normality using Shapiro-Wilks W test, with outliers removed before analyses through the use of box plots. Values were categorized as outliers if their value was greater than 1.5 times the interquartile range, and were then excluded from their data set. Mixed-model ANOVA analyses including the main effects of mitotype, diet, and their interaction were conducted using JMP 13 (SAS institute). Following Aw et al. (2018) if significant main effects were found a *post hoc* two-tailed Student’s *t*-test was conducted. In the case of fecundity, the data were Ln(X+1) transformed prior to analysis. For *N* expression, we conducted a power analyses in JMP to determine the least significant number of samples required for significance. Significance set at 0.05. Unless otherwise stated, all data are biological replicates, which are measurements of biologically distinct samples. Samples size was not predetermined by statistical tests and researchers were not blinded to the studies.

Data used to generate figures is presented as Supplementary Material.

## Results

### Longevity

**FIGURE 1:**
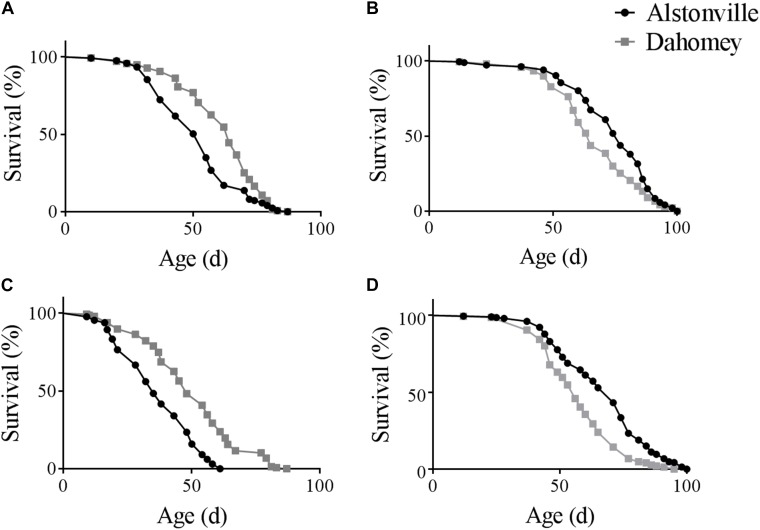
Survival curves of Alstonville and Dahomey mitotypes. **(A)** 1:2 P:C and **(B)** 1:16 P:C diets showing longevity of the Alstonville and Dahomey mitotypes in the *w*^1118^ nuclear background (*n* = 240 flies/mitotype/diet). **(C)** 1:2 P:C and **(D)** 1:16 P:C diets showing longevity of the Alstonville and Dahomey mitotypes in the Oregon R nuclear background (*n* = 240 flies/mitotype/diet).

There was a diet dependent flip in the longevity of the two mitotypes in both the *w*^1118^ and Oregon R nuclear genetic backgrounds. Furthermore, as expected, the longevity of Dahomey and Alstonville in both genetic backgrounds was shorter when fed the 1:2 P:C food. Considering the mitotypes in the *w*^1118^ genetic background and fed the 1:2 P:C diet, Dahomey flies reached 50% survival at 63 days, while Alstonville females reached the same mark at 50 days (Figure 1A). Overall, Dahomey flies lived significantly longer than Alstonville flies (χ^2^ = 16.34, *p* < 0.0001). On the 1:16 P:C diet, Dahomey reached 50% survival at 64 days compared to the 75 days for Alstonville (χ^2^ = 8.69, *p* = 0.003) (Figure 1B).

Longevity of the mitotypes in the Oregon R background showed the same trends as observed in the *w*^1118^ nuclear genetic background, but females tended to be ∼20% shorter lived (Figures 1C,D). On the 1:2 P:C diet, Dahomey flies reached 50% survival at 46 days, while those with the Alstonville mitotype reached it at 33 days (Figure 1C). Overall, Dahomey flies lived significantly longer than Alstonville flies (χ^2^ = 61.55, *p* < 0.001). On the 1:16 P:C diet, Dahomey reached 50% survival at 54 days compared to 68 days for Alstonville (χ^2^ = 33.3, *p* < 0.001).

### CAFÉ Assay

#### Early Fecundity

**FIGURE 2:**
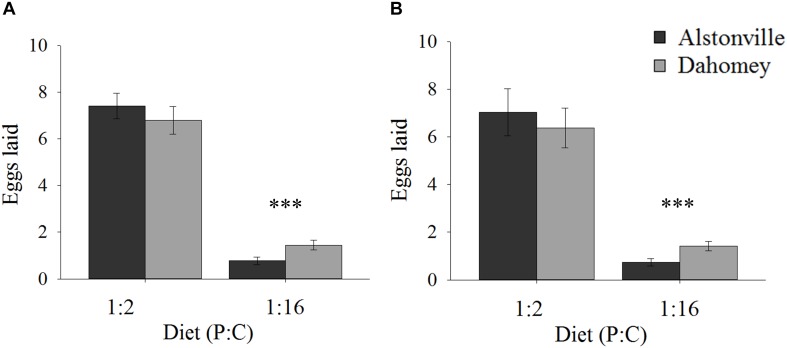
Fecundity. Number of eggs laid by the Alstonville and Dahomey mitotypes fed either the 1:2 or 1:16 P:C diets. **(A)** Females mated with males harboring the same mtDNA type (*n* = 10 flies/mitotype/diet). **(B)**. Females mated with males harboring the *w*^1118^ mitotype (*n* = 10 flies/mitotype/diet). Bars indicate average number of eggs laid over 12 days ± s.e.m. ^∗∗∗^*p* < 0.001 (see text for details).

When fed the 1:2 P:C diet Dahomey laid 8% fewer eggs than Alstonville, but 88% more on the 1:16 P:C diet (Figures 2A,B). ANOVA showed no significant effect of mitotype [*F*_(1,__36)_ = 3.82, *p* = 0.06], but the effect of diet was significant [*F*_(1,__36)_ = 785.55, *p* < 0.0001] and the mitotype by diet interaction was significant [*F*_(1,__36)_ = 20.29, *p* < 0.0001]. The mitotypes did not significantly differ in number of eggs laid on the 1:2 P:C diet [*t*_(18)_ = 0.91, *p* = 0.37] but Dahomey laid significantly more eggs on the 1:16 P:C diet [*t*_(18)_ = 7.60, *p* < 0.0001] (Figure 2A).

Early fecundity showed similar trends when *w*^1118^ was the male mitotype. Dahomey produced 9% fewer eggs on the 1:2 P:C diet but produced 78% more eggs on the 1:16 P:C diet (Figure 2B). ANOVA showed significant main effects of mitotype [*F*_(1,__36)_ = 4.96, *p* = 0.03], diet [*F*_(1,__36)_ = 378.68, *p* < 0.0001] and the mitotype by diet interaction [*F*_(1,__36)_ = 8.87, *p* = 0.005]. The mitotypes did not significantly differ in number of eggs laid on the 1:2 P:C diet [*t*_(18)_ = 0.44, *p* = 0.67] but Dahomey laid significantly more eggs on the 1:16 P:C diet [*t*_(18)_ = 5.09, *p* < 0.0001) (Figure 2B).

#### Feeding

**FIGURE 3:**
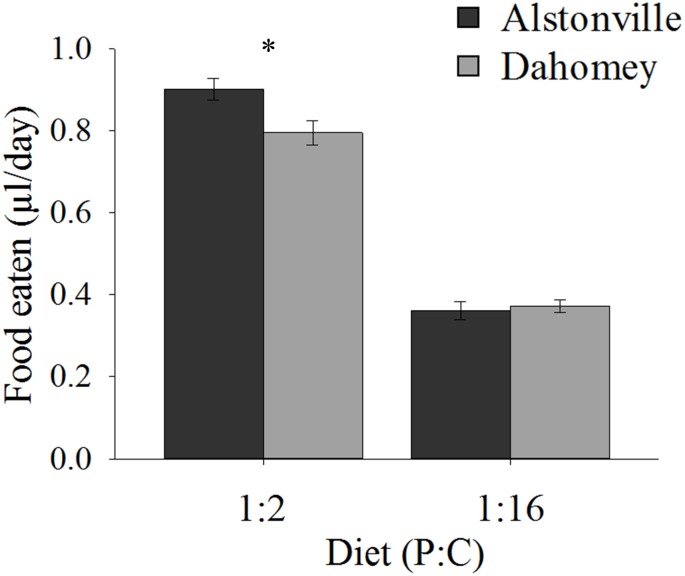
Food eaten. Volume of food consumed by the Alstonville and Dahomey mitotypes fed either the 1:2 or 1:16 P:C diets (*n* = 10 flies/mitotype/diet). Bars indicate average volume of food eaten over 12 days ± s.e.m. ^∗^*p* < 0.05 (see text for details).

When fed the 1:2 P:C diet Dahomey ate 12% less food than Alstonville, but <3% more on the 1:16 P:C diet (Figure 3). ANOVA showed no significant effect of mitotype [*F*_(1,__36)_ = 3.46, *p* = 0.07], but did show significant effects of diet [*F*_(1,__36)_ = 372.80, *p* < 0.0001] and a mitotype by diet interaction [*F*_(1,__36)_ = 5.67, *p* = 0.02]. Dahomey females consumed significantly less than Alstonville adults on the 1:2 P:C diet [*t*_(18)_ = 2.53, *p* = 0.02], but there was no significant difference between mitotypes fed the 1:16 P:C diet [*t*_(18)_ = 0.48, *p* = 0.64] (Figure 3).

**FIGURE 4:**
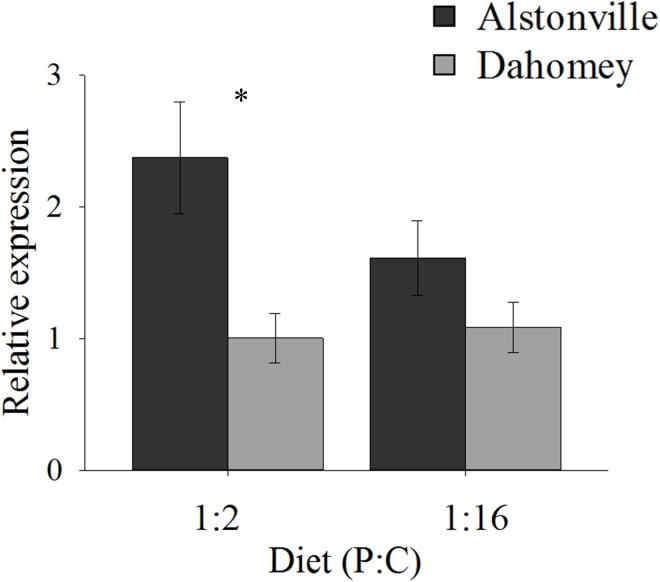
*N* expression. Expression of *N* in the Alstonville and Dahomey mitotypes fed the 1:2 or 1:16 P:C diet (*n* = 6 replicates/mitotype/diet with one outlier removed). Bars indicate relative expression ± s.e.m. ^∗^*p* < 0.05 (see text for details).

*N* expression showed a similar pattern to food consumption. In comparison to Alstonville, Dahomey had 60% lower expression of *N* on the 1:2 P:C diet and 40% lower on the 1:16 P:C diet (Figure 4). ANOVA showed a significant effect of mitotype [*F*_(1,__19)_ = 8.55, *p* = 0.01], but no significant effect of diet [*F*_(1,__19)_ = 1.10, *p* = 0.31] or mitotype by diet interaction [*F*_(1,__19)_ = 1.69, *p* = 0.21]. Dahomey had significantly lower expression of *N* on the 1:2 P:C diet [*t*_(10)_ = 2.7, *p* = 0.02], but the difference was not significant when fed the 1:16 P:C diet [*t*_(9)_ = 1.35, *p* = 0.21]. To further investigate the 1:16 P:C result we conducted a power analysis. Power analysis indicates that a sample size of 26 would be required to show a significant difference (*p* < 0.05) between the mitotypes, if one existed (Σ = 0.64, Δ = 0.26). Therefore, we conclude that there is no biologically distinct difference between *N* expression of flies harboring the two mitotypes when fed the 1:16 P:C diet.

### Physical Activity

#### Walking Speed

**FIGURE 5:**
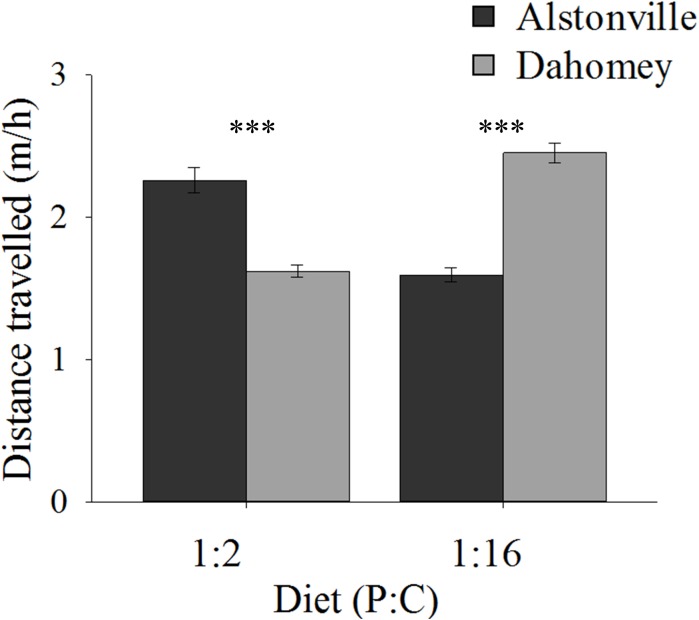
Walking speed. Walking speed of the Alstonville and Dahomey mitotypes fed either the 1:2 P:C or 1:16 P:C diets (*n* = 16 flies/mitotype/diet). Eight flies died during the study: two Alstonville on the 1:2 P:C, three Alstonville on the 1:16 P:C and three Dahomey on the 1:16 P:C diet. Bars indicate distance traveled in m/h ± s.e.m. ^∗∗∗^*p* < 0.001 (see text for details).

On both diets, walking speed was highest in the shorter-lived females suggesting an evolutionary trade-off. Dahomey flies fed the 1:2 P:C diet moved 29% less than Alstonville but moved 44% more on the 1:16 P:C diet (Figure 5). ANOVA of activity showed no significant effect of mitotype [*F*_(1,__52)_ = 0.43, *p* = 0.52] or diet [*F*_(1,__52)_ = 0.02, *p* = 0.90], but showed a significant mitotype by diet interaction [*F*_(1,__52)_ = 20.70, *p* < 0.0001]. Dahomey moved significantly less on the 1:2 P:C [*t*_(28)_ = 3.28, *p* = 0.003], but significantly more on the 1:16 P:C diet [*t*_(24)_ = 3.46, *p* = 0.002].

#### Climbing Ability

**FIGURE 6:**
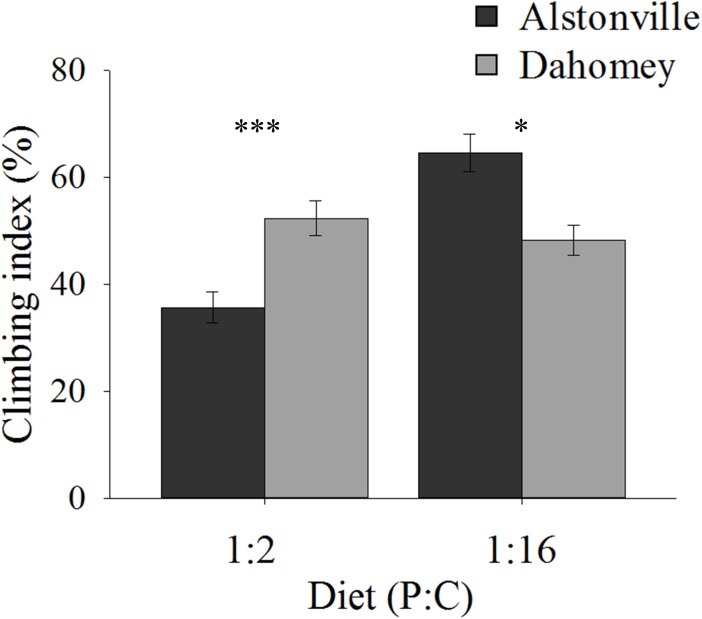
Climbing index. Climbing index of the Alstonville and Dahomey mitotypes fed either the 1:2 or 1:16 P:C diets (*n* = 6 replicates/mitotype/diet). Bars indicate climbing index ± s.e.m. ^∗^*p* < 0.05, ^∗∗∗^*p* < 0.001 (see text for details).

Climbing ability showed the same trend as the longevity data. When fed the 1:2 P:C diet 34% more Dahomey flies climbed above the 80 mm line on the wall of their tubes after negative geotaxis, but 27% fewer climbed to this level on the 1:16 P:C diet (Figure 6). ANOVA of climbing ability did not show a significant main effect of mitotype [*F*_(1,__20)_ = 0.32, *p* = 0.58] or diet [*F*_(1,__20)_ = 1.47, *p* = 0.24], but there was a significant mitotype by diet interaction [*F*_(1,__20)_ = 8.95, *p* = 0.007]. Dahomey had significantly greater climbing ability on the 1:2 P:C diet [*t*_(10)_ = 3.28, *p* = 0.008], but lower ability on the 1:16 P:C diet [*t*_(10)_ = 2.48, *p* = 0.03].

### Mitochondrial Functions

#### RCR, mtDNA Copy Number, and mTerF3 Expression

**FIGURE 7:**
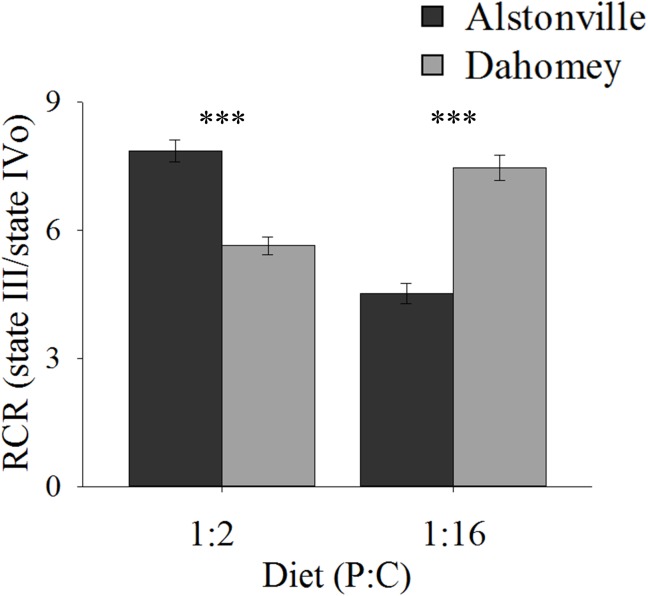
Respiratory control ratio of isolated mitochondria. RCR of the Alstonville and Dahomey mitotypes fed either the 1:2 P:C or 1:16 P:C diet (*n* = 6 replicates/mitotype/diet). Bars indicate RCR ± s.e.m. ^∗∗∗^*p* < 0.001 (see text for details).

RCR showed the same trend as walking-speed. In comparison to Alstonville, RCR was 31% lower in Dahomey flies fed the 1:2 P:C diet but 75% higher when fed the 1:16 P:C food (Figure 7). ANOVA of RCR showed no significant main effect of mitotype [*F*_(1,__20)_ = 2.18, *p* = 0.16] but diet was significant [*F*_(1,__20)_ = 17.76, *p* = 0.0004]. There was a significant interaction between mitotype and diet [*F*_(1,__20)_ = 101.50, *p* < 0.0001]. Dahomey had significantly lower RCR on the 1:2 P:C diet [*t*_(10)_ = 6.78, *p* < 0.0001], but significantly higher RCR on the 1:16 P:C diet [*t*_(10)_ = 7.47, *p* < 0.0001] (Figure 7).

**FIGURE 8:**
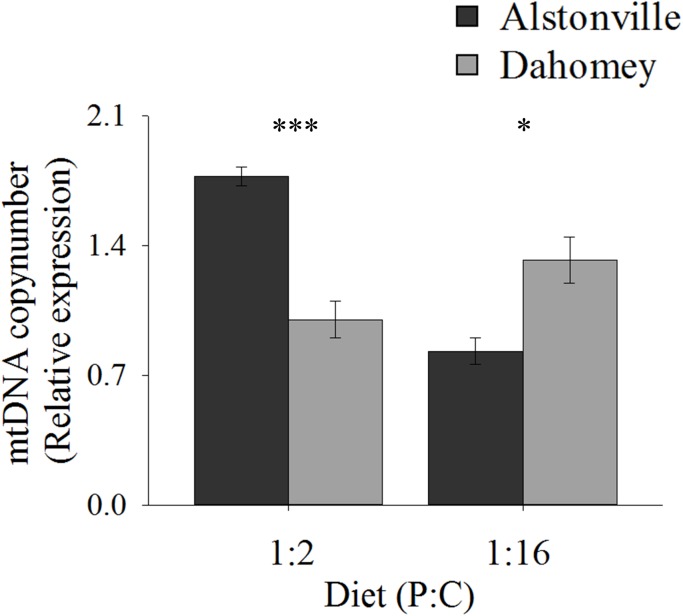
mtDNA copy number. Mitochondrial DNA copy number of the Alstonville and Dahomey mitotypes fed either the 1:2 or 1:16 P:C diet (*n* = 6 replicates/mitotype/diet). Bars indicate relative expression ± s.e.m. ^∗^*p* < 0.05, ^∗∗∗^*p* < 0.001 (see text for details).

Copy number exhibited the same trend as walking-speed and RCR. Dahomey had 50% lower copy number than Alstonville on the 1:2 P:C diet but had 30% higher copy number on the 1:16 P:C diet (Figure 8). ANOVA of mtDNA copy number showed no effect of mitotype [*F*_(1,__20)_ = 1.98, *p* = 0.18], but showed a significant effect of diet [*F*_(1,__20)_ = 9.76, *p* = 0.01], and a mitotype by diet interaction [*F*_(1,__20)_ = 40.36, *p* < 0.0001]. Dahomey had significantly lower copy number on the 1:2 P:C diet [*t*_(10)_ = 6.37, *p* < 0.0001], but had significantly higher copy number on the 1:16 P:C diet [*t*_(10)_ = 3.12, *p* = 0.01] (Figure 8).

**FIGURE 9:**
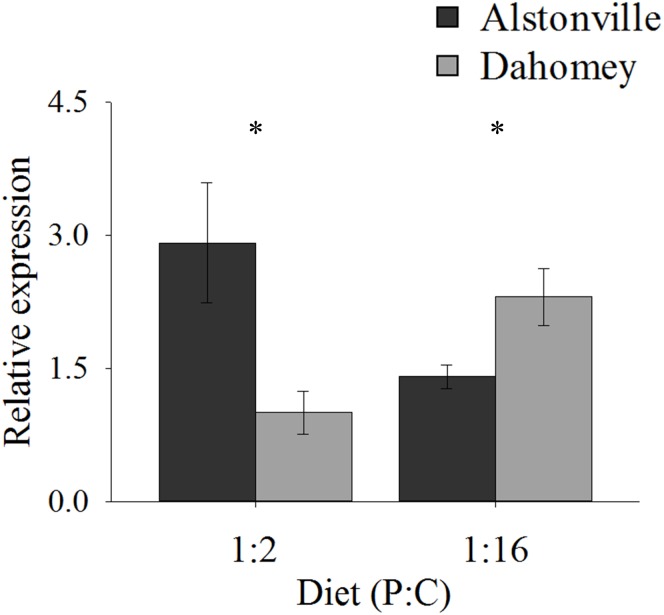
Expression of *mTerF3*. Expression of *mTerF3* in the Alstonville and Dahomey mitotypes fed either the 1:2 or 1:16 P:C diets (*n* = 6 replicates/diet/mitotype). Bars indicate relative expression ± s.e.m. ^∗^*p* < 0.05 (see text for details).

As predicted, the expression of *mTerf3* correlated with copy number and was positively correlated with walking-speed and RCR. Dahomey had 55% lower expression of *mTerf3* than did Alstonville on the 1:2 P:C diet, but 52% higher expression on the 1:16 P:C diet (Figure 9). ANOVA of *mTerf3* expression showed no significant main effect of mitotype [*F*_(1,__20)_ = 1.34, *p* = 0.26], or diet [*F*_(1,__20)_ = 0.05, *p* = 0.82], but showed a significant mitotype by diet interaction [*F*_(1,__20)_ = 10.32, *p* = 0.004]. Dahomey had significantly lower *mTerf3* expression on the 1:2 P:C diet [*t*_(10)_ = 2.41, *p* = 0.04], and significantly higher expression on the 1:16 P:C diet [*t*_(10)_ = 2.36, *p* = 0.04] (Figure 9).

#### Basal ROS and the Antioxidant Response

**FIGURE 10:**
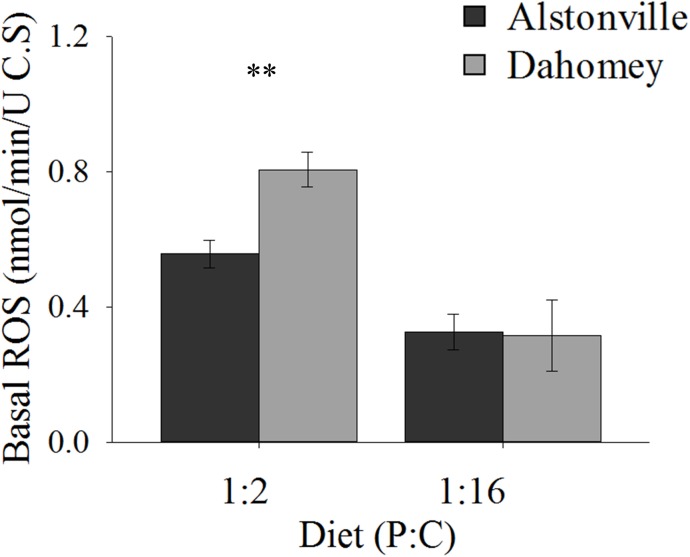
Basal ROS. Basal ROS production of the Alstonville and Dahomey mitotypes fed the 1:2 or 1:16 P:C diets (*n* = 6 replicates/mitotype/diet). Bars indicate average basal ROS production in nmol/min/unit of citrate synthase ± standard error. ^∗∗^*p* < 0.01 (see text for details).

Basal ROS levels were 45% higher in the longer lived Dahomey flies than the shorted lived Alstonville flies when the mitotypes were fed the 1:2 P:C diet (Figure 10). There was no obvious difference in ROS levels between mitotypes when they were fed the 1:16 P:C diet (Figure 10). ANOVA of basal ROS showed significant effects of mitotype [*F*_(1,__20)_ = 21.65, *p* = 0.0002], diet [*F*_(1,__20)_ = 130.09, *p* < 0.0001] and mitotype by diet interaction [*F*_(1,__20)_ = 12.59, *p* = 0.002]. Dahomey had significantly higher basal ROS on the 1:2 P:C diet [*t*_(10)_ = 5.85, *p* = 0.002], but showed no difference on the 1:16 P:C die [*t*_(10)_ = 0.7742, *p* = 0.46].

**FIGURE 11:**
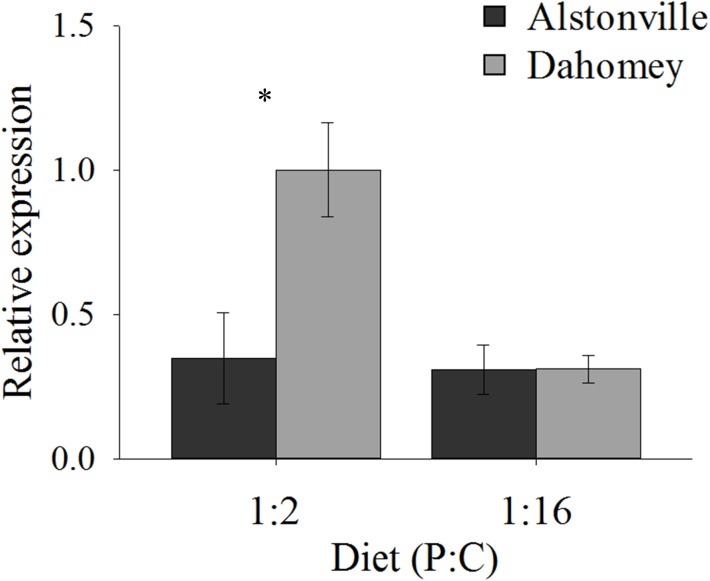
Expression of *GstE1*. Expression of *GstE1* in the Alstonville and Dahomey mitotypes fed either the 1:2 or 1:16 P:C diet (*n* = 6 replicates/mitotype/diet). Bars indicate relative expression ± s.e.m. ^∗^*p* < 0.05 (see text for details).

As expected, *GstE1* expression showed the same trend as basal ROS. Dahomey has 180% higher expression of *Gste1* than Alstonville on the 1:2 P:C diet (Figure 11). There was no obvious difference in expression in flies fed the 1:16 P:C diet (Figure 11). ANOVA of *GstE1* expression showed significant main effects of mitotype [*F*_(1,__20)_ = 5.92, *p* = 0.02] and diet [*F*_1,__20)_ = 7.30, *p* = 0.01], and a significant mitotype by diet interaction [*F*_(1,__20)_ = 5.83, *p* = 0.03]. Expression of *GstE1* was significantly higher in Dahomey on the 1:2 P:C diet [*t*_(10)_ = 2.64, *p* = 0.02], but showed no difference on the 1:16 P:C diet [*t*_(10)_ = 0.02, *p* = 0.98].

## Discussion

MtDNA has been used extensively as a tool for inferring the evolutionary and demographic past of populations and species and is often presumed to evolve in a manner consistent with a strictly neutral equilibrium model without testing this assumption (Ballard and Kreitman, 1995; Ballard and Whitlock, 2004). This assumption can no longer be supported (Bazin et al., 2006; Galtier et al., 2009; James et al., 2016; Teske et al., 2018). Here we show that an interaction between dietary macronutrient ratio and mitotype influences a wide variety of life history traits and mitochondrial functions. Currently, it is not clear how common such events may be in nature but the results of James et al. (2016) suggest they may be quite pervasive. James et al. (2016) results indicate that up to 60% of non-synonymous substitutions could be fixed by positive selection in invertebrates and may therefore have a non-trivial impact on mitochondrial diversity. The important consequence of their results is that mtDNA diversity may reflect the amount of time since the last selective sweep, rather than strictly demographic processes that affect the population, which may then affect tests of isolation by distance (Teske et al., 2018).

To examine the data gathered we first consider the differences in longevity between the mitotypes fed the 1:2 P:C and 1:16 P:C diets. Remarkably, when both nuclear genetic backgrounds are considered, Dahomey lived, on average, 21% longer on the 1:2 P:C diet, but perished 10% earlier on the 1:16 P:C diet. Further, as previously reported, females lived longer on the 1:16 P:C food than the 1:2 P:C diet (Lee et al., 2008). Clancy (2008) previously assayed the longevity of these mitotypes in the *w*^1118^ genetic background and found that the Dahomey had a 4% reduction in lifespan on an unknown diet. Aw et al. (2017) studied the longevity of Alstonville females in vials and observed similar results to those obtained here. They found 50% survival increased from 48 days on a 1:2 P:C diet to 75 days on a 1:16 P:C diet.

Here, we observed that climbing ability was correlated with longevity. Bazzell et al. (2013) measured climbing ability of the Canton S and Berlin K strains through automated negative geotaxis. They found that the Berlin K line had 3 times lower climbing ability on a 1:4 P:C diet, but had greater climbing ability on a 1:2 P:C diet. Mawhinney and Staveley (2011) studied the climbing ability and longevity of *Drosophila* that differed in expression of green fluorescent protein. They demonstrated a positive correlation between 50% survival and climbing ability.

Showing the same trend as longevity and climbing levels of ROS and expression of *GstE1* were higher in Dahomey flies on the 1:2 P:C diet. There, however, were no obvious differences between mitotypes when flies were fed the 1:16 P:C diet. Aw et al. (2017) assayed maximum ROS production from 11 days old Alstonville females and females with the Japan mitotype (in the *w*^1118^ nuclear background) that were fed a range of P:C diets. They found maximum ROS was highest when fed the 1:2 and 1:16 P:C diet, and lowest when fed a 1:8 P:C diet. Mitotypes did not significantly differ in maximal ROS production, and basal ROS was not measured. *GstE1* expression was measured as an indicator of antioxidant capacity. High antioxidant capacity is indicative of high longevity (Ng et al., 2014) by minimizing cytotoxic damage (Luceri et al., 2018). Expression of *GstE1* has been shown to have increased twofold in *yw* *Drosophila* under expressing peroxiredoxins compared to the control at 13 days of age (Odnokoz et al., 2017) and in strains that show differential expression of antimicrobial peptides (Zhao et al., 2011). We hypothesize that the difference in basal levels of ROS that we observed in flies fed the 1:2 P:C diet resulted in mitohormetic responses from the nuclear genome. These responses include an upregulation of *GstE1* expression may have provided an advantage through mitohormesis (Ristow and Schmeisser, 2014; Schaar et al., 2015). Mitohormesis has been shown promote longevity in *Drosophila* through microbiome remodeling (Obata et al., 2018) and repression of insulin signaling (Owusu-Ansah et al., 2013).

Here we observed that walking-speed was inversely correlated with longevity. Further, walking-speed was positively correlated with the mitochondrial functions of RCR, mtDNA copy number and *mTerF3* expression. Differences in walking speed between females harboring different mitotypes have not been reported, however, macronutrient ratio of diet has been found to influence walking speed in *Drosophila* (Catterson et al., 2010). Their data showed consistently higher movement on diet that contained only sucrose, in comparison to diet with sucrose and yeast, in *w*^1118^ females. In terms of mitochondrial functions, Pichaud et al. (2010) found that male *Drosophila simulans* harboring distinct mitotypes (*si*II and *si*III) differed significantly in RCR due to temperature but they did not test the influence of diet. For copy number, Aw et al. (2017) did not detect any significant difference in mtDNA copy number in 11 days old Japan or Alstonville females when they were fed any of the P:C diets tested. The Japan mitotype differs from Alstonville by a single non-synonymous mutation, whereas Dahomey differs from Alstonville by three non-synonymous substitutions. Zhu et al. (2014) saw that the w501 mitotype had 50% higher mtDNA copy number than sm21 in the Oregon R nuclear background. As predicted *mTerF3* expression was positively correlated with mtDNA copy number. The mitoribosome synthesizes all mitochondrial encoded proteins (Richman et al., 2014) and mutations in the mitoribosome have been linked to specific diseases (Elson et al., 2015). Plausibly, differential function of the mitoribosome influenced the number of mitochondrial Complexes, which resulted in the observed difference in OXPHOS efficiency.

Consistent with the walking speed result on the 1:2 P:C diet, feeding and expression of *N* are downregulated in Dahomey. This suggests that the observed decrease in mitochondrial function in Dahomey is signaling to reduce feeding. Feeding rate is a direct measure of energy intake of an organism and is influenced by a range of factors in *Drosophila* including gustatory systems, energy sensors and protein sensors (Wu et al., 2005; Vargas et al., 2010; Fujita and Tanimura, 2011). An alternate explanation for the reduced feeding rate in Dahomey flies fed the 1:2 P:C diet is that their ability to feed from capillaries reduced over time. Bajracharya and Ballard (2016) found that flies with Parkinson’s phenotype had reduced walking speed and could not easily feed during CAFÉ assay.

Dahomey had 78% higher fecundity than Alstonville when fed the 1:16 P:C diet, but fecundity did not differ between flies fed the 1:2 P:C food. As previously reported fecundity in both mitotypes was higher when fed the 1:2 P:C food than fed the 1:16 P:C diet (Lee et al., 2008). This result implies a trade-off with longevity on the 1:16 P:C but not the 1:2 P:C diet as fecundity as a trade-off has been shown to occur under stressful conditions (Beaulieu et al., 2015). Further studies investigating the mechanism underlying this evolutionary trade-off between these mitotypes are warranted. It has already been shown that Dahomey has higher fecundity than several wild-caught strains including flies sourced from France, Germany, and Greece when fed diet that differed in yeast concentration (Metaxakis and Partridge, 2013).

Aw et al. (2018) studied these same strains in larvae and provided compelling evidence to suggest that the ND4 (V161L) mutation in Dahomey is driving differences in larval development time. Here we hypothesize that the same V161L mutation also drives the observed differences in adults. However, additional studies are required to definitively show no linked mtDNA mutations are functionally significant and determine whether the same metabolic pathways opperate in larvae and adults.

In this study, we have identified significant differences in organismal physiology and mitochondrial functions in Dahomey and Alstonville females. Notably, Dahomey females live longer than Alstonville flies on a high protein diet but are shorter lived on a high carbohydrate diet. This flip in longevity is inversely correlated with a battery of mitochondrial functions suggesting an evolutionary-trade off. We did not see a trade-off between longevity and fecundity within each diet. Rather, fecundity only differed on the high carbohydrate 1:16 P:C diet. These results suggest that diet may be an important driver of mtDNA dynamics and suggest future studies explore the genetic variation within and among populations feeding on divergent foods.

## Data Availability

The raw data supporting the conclusions of this manuscript will be made available by the authors, without undue reservation, to any qualified researcher.

## Author Contributions

The study was designed by ST and JB. ST conducted all assays, collected all the data, and analyzed all the data. ST wrote the first draft of the manuscript, and JB contributed substantially to revisions.

## Conflict of Interest Statement

The authors declare that the research was conducted in the absence of any commercial or financial relationships that could be construed as a potential conflict of interest.

## Supplementary Material

Table S1 Data used to generate figures.
